# Retraction Note: Overexpression of MAP3K3 promotes tumour growth through activation of the NF-κB signalling pathway in ovarian carcinoma

**DOI:** 10.1038/s41598-025-20904-y

**Published:** 2025-09-23

**Authors:** Ying Zhang, Sha-Sha Wang, Lin Tao, Li-Juan Pang, Hong Zou, Wei-Hua Liang, Zheng Liu, Su-Liang Guo, Jin-Fang Jiang, Wen-Jie Zhang, Wei Jia, Feng Li

**Affiliations:** 1 Department of Pathology and Key Laboratory of Xinjiang Endemic and Ethnic Diseases, Shihezi University School of Medicine, Shihezi, 832002 Xinjiang China; 2 Department of Pathology and Medical Research Center, Beijing Chaoyang Hospital, Capital Medical University, Beijing, 100020 China

Retraction of: *Scientific Reports* 10.1038/s41598-019-44835-7, published on 10 June 2019

The Editors have retracted this Article. After the publication of this Article, it was brought to the Editors’ attention that several panels, or portions of panels, in Figs. 2A, B, and C, Figs. 2D, E, and F, and Fig. 2G appear to overlap with one another. In addition, two panels in Fig. 3J, two gel slices in Fig. 5A, and two portions of panels in Fig. 5B also appear to overlap with each other.

The Editors therefore no longer have confidence in the results reported in the Article.

None of the Authors responded to the correspondence from the Editors about this retraction.

